# *BLM* mutation is associated with increased tumor mutation burden and improved survival after immunotherapy across multiple cancers

**DOI:** 10.1002/cam4.6716

**Published:** 2023-12-20

**Authors:** Huiping Shi, Liang Gao, Hong Yin, Min Jiang

**Affiliations:** ^1^ The First Affiliated Hospital of Soochow University Suzhou PR China; ^2^ Institutes of Biology and Medical Sciences Soochow University Suzhou PR China; ^3^ Department of Oncology The First Affiliated Hospital of Soochow University Suzhou PR China

**Keywords:** *BLM* mutation, immunotherapy, survival, tumor mutation burden

## Abstract

**Background:**

*BLM* encodes a RecQ DNA helicase that regulates genomic stability, and its mutations are associated with increased cancer susceptibility. Here, we show a multifaceted role of *BLM* mutations in tumorigenesis and immunotherapy.

**Methods and Results:**

A total of 10,967 cancer samples from the cancer genome atlas database were analyzed, 1.6% of which harbored *BLM* somatic mutations. *BLM* mutation was found to be associated with increased tumor mutation burden and more immune‐active tumor microenvironment in these patients. Moreover, clinical data of 2785 patients from nine immunotherapy studies were analyzed to study *BLM* mutations' impact on immunotherapy. Among them, 69 patients harbored *BLM* mutations, and interestingly, they had significantly higher survival probability than patients without *BLM* mutations. Cancer patients with *BLM* mutations had higher complete response and partial response rates, but lower progressive disease rate than *BLM* nonmutant patients.

**Conclusion:**

Our study shows that *BLM* mutation is related to improved survival after immunotherapy across multiple cancers.

## 1 BACKGROUND

The *BLM* gene encodes bloom syndrome protein (BLM), which is one of the RecQ DNA helicases. It participates in DNA replication and repair by unwinding paired DNA. *BLM* mutations can lead to genomic instability and increase cancer susceptibility,^1^ which is the pathological mechanism of the autosomal recessive human disease called bloom syndrome. Emerging evidence indicates that *BLM* mutation participates in the occurrence of multiple cancers, such as colorectal cancer,^2^ mesothelioma,^3^ and prostate cancer.^4^ Some studies also indicate *BLM* overexpression as an adverse prognostic factor in cancer patient survival.^5^

Immune checkpoint inhibitors have been developed to reinvigorate T‐cell cytotoxicity and enhance cancer cell killing. Immunotherapy provides remarkable benefits in some cancer patients, but a considerable number of recipients fail to respond. The prediction of immunotherapy efficacy remains a problem. Tumor mutation burden (TMB) is the rate of genetic mutations in a cancer sample. A higher TMB has been found to be associated with more immunogenic neoantigens, stronger anti‐tumor immune reaction and increased sensitivity to immunotherapy in some cancer types, such as melanoma and lung cancer.^6^

We analyzed 10,967 cancer samples from the cancer genome atlas (TCGA) database and found that *BLM* mutation was associated with increased TMB and more immune‐active tumor microenvironment. Moreover, a total of 2785 patients from nine immunotherapy studies were analyzed to reveal the role of *BLM* mutation in immunotherapy efficacy, and the results showed that *BLM* mutation was related to improved survival after immunotherapy across multiple cancers.

## 2 RESULTS AND DISCUSSION

**FIGURE 1:**
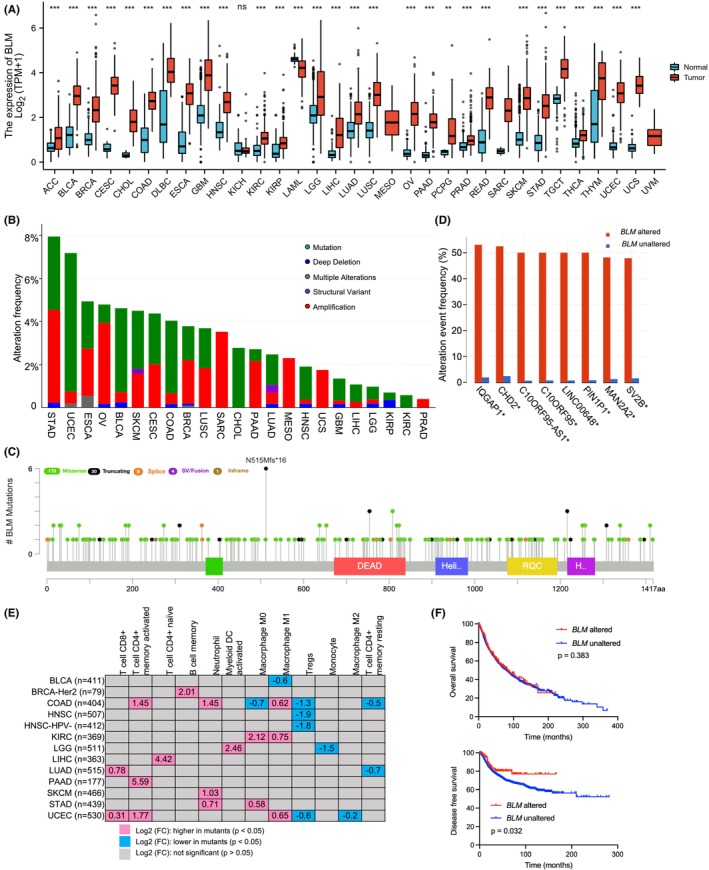
*BLM* mutation spectrum in multiple cancer types and its association with tumor immune landscape. (A) Comparison of *BLM* expression levels between cancer patients and healthy controls. Data were presented as median with its interquartile range. (B) *BLM* alteration frequency in multiple TCGA cancer types. (C) Distribution of multiple types of *BLM* mutations. (D) The co‐altered genes in *BLM* altered cancer patients. (E) The infiltration of multiple immune cells in cancer patients with or without *BLM* mutation. (F) Overall survival and disease‐free survival of TCGA cancer patients with or without *BLM* alteration. ACC: adrenocortical carcinoma; BLCA: bladder urothelial carcinoma; BRCA: breast invasive carcinoma; CESC: cervical squamous cell carcinoma; CHOL: cholangiocarcinoma; COAD: colon adenocarcinoma; DLBC: diffuse large B cell lymphoma; ESCA: esophageal carcinoma; GBM: glioblastoma multiforme; HNSC: head and neck squamous cell carcinoma; KICH: kidney chromophobe; KIRC: kidney renal clear cell carcinoma; KIRP: kidney renal papillary cell carcinoma; LAML: acute myeloid leukemia; LGG: brain lower grade glioma; LIHC: liver hepatocellular carcinoma; LUAD: lung adenocarcinoma; LUSC: lung squamous cell carcinoma; MESO: mesothelioma; OV: ovarian serous cystadenocarcinoma; PAAD: pancreatic adenocarcinoma; PCPG: pheochromocytoma and paraganglioma; PRAD: prostate adenocarcinoma; READ: rectum adenocarcinoma; SARC: sarcoma; SKCM: skin cutaneous melanoma; STAD: stomach adenocarcinoma; TGCT: testicular germ cell tumors; THCA: thyroid carcinoma; THYM: thymoma; UCEC: uterine corpus endometrial carcinoma; UCS: uterine carcinosarcoma; UVM: uveal melanoma.

First, *BLM* expression levels were compared between cancer patients and healthy controls, and we found that *BLM* expression was significantly upregulated in most cancer types (Figure 1A). *BLM* expression was correlated with patient survival in multiple cancer types (Figure S1). This effect on survival was at least partially attributed to its regulation of tumor immune landscape, as shown by the significant differences in immune cell infiltration in multiple tumors with high or low *BLM* expression (Figure S2). To determine the role *BLM* mutation in cancer, we analyzed the genetic and clinical data of a total of 10,967 samples from TCGA database. We found that 311 (3%) of the samples had *BLM* alterations (including mutation, copy number change and structural variant). Cancer types with the highest frequency of *BLM* alteration were stomach adenocarcinoma (7.95%) and uterine corpus endometrial carcinoma (7.18%) (Figure 1B) The compositions of cancer samples with or without *BLM* alterations are shown in Figure S3. The BLM protein has Helicase_C, RecQ carboxyl‐terminal (RQC), helicase and RNase D C‐terminal (HRDC), DEAD (domain with Asp (D)‐Glu (E)‐Ala (A)‐Asp (D)/His sequence), and Bloom's syndrome DEAD helicase C‐terminal (BDHCT) domains, but *BLM* mutations were found throughout its whole sequence, with missense mutation being the commonest, followed by truncating mutation (Figure 1C). The genes with the highest co‐alteration rates with *BLM* mutation were shown in a bar graph (Figure 1D). Interestingly, *BLM* copy number was associated with immune cell infiltration (Figure S4), immunomodulators and chemokine levels (Figure S5). Importantly, *BLM* mutations also significantly affected the immune landscape of cancer patients (Figure 1E). Intriguingly, the results showed that the infiltration of CD8^+^ T cells, activated CD4^+^ memory T cells, naïve CD4^+^ T cells, memory B cells, neutrophils, and activated myeloid dendritic cells were significantly increased in cancer patients with *BLM* mutations when compared to those without *BLM* mutations. On the other hand, the infiltration levels of Tregs, M2 macrophages and resting CD4^+^ memory T cells were significantly lower in cancer patients with *BLM* mutations. These results indicate that the cancer patients with *BLM* mutations have a more immune‐active tumor microenvironment, which may be related to enhanced immunotherapy efficacy.

**FIGURE 2:**
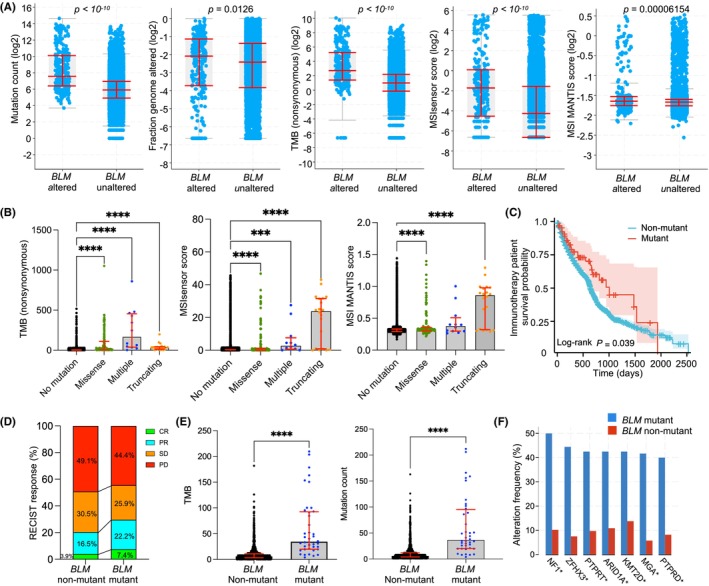
The association of *BLM* mutation with tumor mutation burden (TMB) and survival after immunotherapy. (A) Comparisons of mutation count, altered genome fraction, TMB, microsatellite instability (MSI) sensor score and MSI Microsatellite Analysis for Normal‐Tumor InStability (MANTIS) score in *BLM* altered group and unaltered group. Data were presented as median with its interquartile range. (B) Comparisons of TMB, MSIsensor score and MSI MANTIS score in *BLM* nonmutant patients and patients with *BLM* missense, truncating or multiple mutations. Data were presented as median with its interquartile range. (C) Survival outcome of *BLM* mutant and nonmutant cancer patients treated with immune checkpoint inhibitors in the nine studies. (D) Fractions of immunotherapy‐treated cancer patient response as classified by the Response Evaluation Criteria in Solid Tumors (RECIST) guideline (CR: complete response, PR: partial response, SD: stable disease, PD: progressive disease). (E) Comparisons of TMB and mutation count in *BLM* mutant and nonmutant cancer patients from a multi‐cancer cohort (study 1). Data were presented as median with its interquartile range. (F) Top co‐altered genes in *BLM* mutant cancer patients from a multi‐cancer cohort (study 1).

To characterize *BLM* mutation in cancer and its association with TMB, we analyzed a total of 10,967 samples from TCGA database and found that 1.6% of the samples harbored *BLM* somatic mutations. The samples were categorized into *BLM* altered and *BLM* unaltered groups. The overall survival was not different between the two groups, but the disease‐free survival probability was significantly higher in the altered group (Figure 1F). We found that the mutation count, altered genome fraction, TMB, microsatellite instability (MSI) sensor and MSI Microsatellite Analysis for Normal‐Tumor InStability (MANTIS) score were significantly higher in the *BLM* altered group, indicating that *BLM* mutation contributes to genomic instability and higher TMB (Figure 2A). Compared to cancer patients with no mutation, those with missense mutations, truncating mutations or multiple mutations had significantly higher TMB and MSIsensor score (Figure 2B). Cancer patients with missense mutations or truncating mutations had significantly higher MSI MANTIS score when compared to nonmutant patients (Figure 2B).

To unravel the relationship between *BLM* mutation and survival after immunotherapy, we reviewed the literature and summarized studies in which cancer patients were treated with immune checkpoint inhibitors and sequenced for genomic mutations (whole exome or targeted sequencing). A total of nine studies were included in the analysis^6^, ^7^, ^8^, ^9^, ^10^, ^11^, ^12^, ^13^, ^14^, ^15^ and the information was summarized in Table S1. Monotherapy or combination therapy with anti‐programmed death‐1 (PD‐1), anti‐programmed cell death ligand 1 (PD‐L1), and anti‐cytotoxic T‐lymphocyte associated protein 4 (CTLA4) antibodies were administered in these cancer patients. In total, there were 2785 patients with sufficient data for analysis, and 69 (2.5%) of them harbored *BLM* mutations. We compared the survival probability of immunotherapy‐treated cancer patients with or without *BLM* mutations. Intriguingly, cancer patients without *BLM* mutations had significantly reduced survival probability when compared to those with *BLM* mutations (hazard ratio = 1.497 (95% confidence interval: 1.086–2.062), *p* = 0.039) (Figure 2C). Patient response was classified into four sub‐groups according to the Response Evaluation Criteria in Solid Tumors (RECIST) guideline (CR: complete response, PR: partial response, SD: stable disease, PD: progressive disease),^16^ and the immunotherapy response differed in cancer patients with or without *BLM* mutations (Figure 2D). We found that cancer patients with *BLM* mutations had higher CR (7.4% vs. 3.9%) and PR (22.2% vs. 16.5%) rates, but lower PD rate (44.4% vs. 49.1%) than those without *BLM* mutations (Figure 2D). Consistent with the TCGA data, for the 1661 patients in Samstein's study,^6^ those with *BLM* mutations had significantly increased mutation count and TMB than the nonmutant ones (Figure 2E). When the *BLM* gene was mutated, the altered frequencies of *NF1*, *ZFHX3*, *PTPRT*, *ARID1A*, *KMT2D, MGA*, and *PTPRD* were significantly increased (Figure 2F). Additionally, multivariate cox regression analysis was performed to evaluate the predictive role of *BLM* mutation in immunotherapy in a total of 782 patients with their information including survival data, cancer type, *BLM* mutation, and RECIST classification. Multivariate cox regression analysis showed that *BLM* mutation is a positive predictive factor (*p* = 0.038) for survival after immunotherapy in cancer patients, with a hazard ratio of 0.348 (95% confidence interval: 0.129–0.943) (Table S2). These results indicate that cancer patients with *BLM* mutations have higher TMB, improved immunotherapy response and increased survival after immunotherapy than those without *BLM* mutations.

Despite these interesting findings, our study is limited by the possibility of bias and confounding factors, which include co‐existing mutations, additional treatments before, after or during immunotherapy, differing clinical conditions, cancer types, grade, and stage. Proper standardization of the patient data regarding these factors is quite important. In our analysis, we summarized the results from nine immunotherapy studies, which provided varied information of the patients due to their different study goals. For example, only some studies listed Eastern Cooperative Oncology Group (ECOG) performance status and number of metastatic sites, while other studies showed tumor stage and mutation subtype. The heterogeneity of these data made the assembly and standardization of all patient data difficult. Indeed, the response to immunotherapy depends on the cumulative effects of multiple factors. Therefore, we generated a prognostic model that incorporated different factors to improve the prediction of immunotherapy response. Using the data from a total of 853 patients with non‐small cell lung cancer, we performed multivariate cox regression analysis and calculated a risk score for each patient, which was based on baseline age, ECOG performance status, *EML4‐ALK* rearrangement status, and *BLM* mutation. According to this model, non‐small cell lung cancer patients with lower risk scores had higher survival probability than those with higher risk scores (Figure S6). The coefficient of negative *BLM* mutation in the calculation of risk score is 1.1127409987695 (which is >0), indicating that negative *BLM* mutation contributed to higher risk score whereas the existence of *BLM* mutation led to lower risk score and higher survival probability.

## 3 METHODS

### 3.1 Pan‐cancer analysis of *BLM* mutation

The expression of *BLM* was compared between cancer patients and healthy controls using data extracted from TCGA (https://portal.gdc.cancer.gov) and the genotype‐tissue expression (GTEx) databases (https://gtexportal.org/home/). The *BLM* gene alteration frequency, mutation loci, co‐altered genes, patient survival data, mutation count, altered genome fraction, TMB, MSIsensor score and MSI MANTIS score were analyzed using the cBioPortal for Cancer Genomics^17^ (https://www.cbioportal.org/).

### 3.2 Tumor immune landscape analysis

The infiltration of multiple immune cells in cancer patients with or without *BLM* mutation was compared using Tumor IMmune Estimation Resource (TIMER) 2.0 database^18^ (http://timer.cistrome.org/). The infiltration of multiple immune cells in cancer patients with different *BLM* expression levels or copy numbers was analyzed using TIMER database^19^ (https://cistrome.shinyapps.io/timer/). The relationships between *BLM* copy number and the levels of immunomodulators (major histocompatibility complexes, immunoinhibitors, immunostimulators), chemokines and chemokine receptors were compared using tumor immune system interaction database (TISIDB) database^20^ (http://cis.hku.hk/TISIDB/index.php).

### 3.3 Immunotherapy study analysis

A comprehensive literature review was performed to identify clinic trials in which cancer patients were treated with immune checkpoint inhibitors (anti‐PD‐1, anti‐PD‐L1, and/or anti‐CTLA4) and sequenced for genomic mutations using whole exome or targeted next generation sequencing. There are nine eligible studies included in our study as shown in Table S1. The patient survival, immunotherapy efficacy, genomic mutation and TMB data were extracted and compared.

### 3.4 Statistical analysis

Statistical analysis and result visualization was performed using R (version 3.6.3). Survival analysis was performed using the survminer and survival R packages and log‐rank test was performed. Hazard ratio with 95% confidence intervals was reported. Parametric data was analyzed using unpaired two‐sided Student's *t*‐test and nonparametric data was analyzed using Mann–Whitney test in GraphPad Prism. Mutation count, altered genome fraction, TMB, MSIsensor score and MSI MANTIS score were compared using Wilcoxon test. *p* < 0.05 was considered statistically significant.

## 4 CONCLUSION

In summary, we show that the role of *BLM* mutation is multifaced in carcinogenesis and immunotherapy. *BLM* mutation is associated with increased TMB and improved survival after immunotherapy. *BLM* mutation is a potential biomarker for immunotherapy efficacy prediction and patient stratification across multiple cancer types.

## AUTHOR CONTRIBUTIONS

**Huiping Shi:** Conceptualization (equal); data curation (equal); formal analysis (equal); writing – original draft (equal). **Liang Gao:** Conceptualization (equal); data curation (equal); formal analysis (equal); writing – original draft (equal). **Hong Yin:** Conceptualization (equal); funding acquisition (equal); supervision (equal); writing – review and editing (equal). **Min Jiang:** Conceptualization (equal); funding acquisition (equal); supervision (equal); writing – review and editing (equal).

## CONFLICT OF INTEREST STATEMENT

The authors declare no competing interests.

## Supporting information


Data S1.

## Data Availability

The data that support the findings of this study are available from the corresponding author upon reasonable request.
