# Supplementary material for: BLM mutation is associated with increased tumor mutation burden and improved survival after immunotherapy across multiple cancers
Source: Cancer Med. 2023 Dec 20;13(1):e6716. doi: 10.1002/cam4.6716 (PMC10807622; doi:10.1002/cam4.6716)

Table S1. Patient features in the 9 immunotherapy studies

Study	Cancer	Immunotherapy	Number of patients with sufficient data	*BLM* mutation	Number of patients	Alive/dead	Ref
**1**	Multiple cancer types	Anti PD-1†, anti-PD-L1‡, anti-CTLA4§	1661	Negative	1621	805/816	1
				Positive	40	24/16	
**2**	Non-small-cell lung cancer	Anti-PD-L1	425	Negative	420	266/154	2,3
				Positive	5	5/0	
**3**	Non-small-cell lung cancer	Anti-PD-L1	144	Negative	142	88/54	3,4
				Positive	2	2/0	
**4**	Melanoma, lung cancer, bladder cancer, head and neck squamous cell carcinoma, anal cancer, sarcoma	Anti PD-1, anti-PD-L1, anti-CTLA4	249	Negative	238	119/119	5
				Positive	11	6/5	
**5**	Melanoma	Anti-CTLA4	110	Negative	105	26/79	6
				Positive	5	1/4	
**6**	Melanoma	Anti PD-1	68	Negative	65	28/37	7
				Positive	3	3/0	
**7**	Melanoma	Anti PD-1, anti-CTLA4	56	Negative	54	29/25	8
				Positive	2	2/0	
**8**	Melanoma	Anti PD-1	37	Negative	36	18/18	9
				Positive	1	0/1	
**9**	Renal cell carcinoma	Anti PD-1	35	Negative	35	12/23	10
				Positive	0	0/0	

†PD-1: programmed death-1; ‡PD-L1: programmed cell death ligand 1; §CTLA4: cytotoxic T-lymphocyte associated protein 4

Table S2. Multivariate COX regression analysis of factors associated with survival after immunotherapy

Characteristics	Total(N)	Univariate analysis		Multivariate analysis	
		Hazard ratio (95% CI)	P value	Hazard ratio (95% CI)	P value
BLM mutation	782		**0.011**		
Negative	767	Reference		Reference	
Positive	15	0.346 (0.129 - 0.929)	**0.035**	0.348 (0.129 - 0.943)	**0.038**
Cancer type	782		0.090		
Melanoma	178	Reference		Reference	
Renal cell	35	0.771 (0.493 - 1.206)	0.254	0.796 (0.505 - 1.254)	0.325
Non-small-cell lung cancer	569	1.195 (0.931 - 1.534)	0.161	1.221 (0.943 - 1.580)	0.130
RECIST[†]	734		**< 0.001**		
SD[§]	253	Reference		Reference	
PD[‡]	362	1.726 (1.351 - 2.205)	**< 0.001**	1.737 (1.356 - 2.225)	**< 0.001**
PR[¶]	105	0.797 (0.589 - 1.078)	0.141	0.805 (0.594 - 1.090)	0.161
CR[Π]	14	0.429 (0.208 - 0.882)	**0.021**	0.430 (0.208 - 0.888)	**0.023**

[†]RECIST: Response Evaluation Criteria in Solid Tumors; [§]SD: stable disease; [‡]PD: progressive disease; [¶]PR: partial response; [Π]CR: complete response

References

1. Samstein RM, Lee CH, Shoushtari AN, et al. Tumor mutational load predicts survival after immunotherapy across multiple cancer types. *Nat Genet*. 2019;51(2):202-206.
2. Rittmeyer A, Barlesi F, Waterkamp D, et al. Atezolizumab versus docetaxel in patients with previously treated non-small-cell lung cancer (OAK): a phase 3, open-label, multicentre randomised controlled trial. *Lancet*. 2017;389(10066):255-265.
3. Gandara DR, Paul SM, Kowanetz M, et al. Blood-based tumor mutational burden as a predictor of clinical benefit in non-small-cell lung cancer patients treated with atezolizumab. *Nat Med*. 2018;24(9):1441-1448.
4. Fehrenbacher L, Spira A, Ballinger M, et al. Atezolizumab versus docetaxel for patients with previously treated non-small-cell lung cancer (POPLAR): a multicentre, open-label, phase 2 randomised controlled trial. *Lancet*. 2016;387(10030):1837-1846.
5. Miao D, Margolis CA, Vokes NI, et al. Genomic correlates of response to immune checkpoint blockade in microsatellite-stable solid tumors. *Nat Genet*. 2018;50(9):1271-1281.
6. Van Allen EM, Miao D, Schilling B, et al. Genomic correlates of response to CTLA-4 blockade in metastatic melanoma. *Science*. 2015;350(6257):207-211.
7. Riaz N, Havel JJ, Makarov V, et al. Tumor and Microenvironment Evolution during Immunotherapy with Nivolumab. *Cell*. 2017;171(4):934-949 e916.
8. Roh W, Chen PL, Reuben A, et al. Integrated molecular analysis of tumor biopsies on sequential CTLA-4 and PD-1 blockade reveals markers of response and resistance. *Sci Transl Med*. 2017;9(379).
9. Hugo W, Zaretsky JM, Sun L, et al. Genomic and Transcriptomic Features of Response to Anti-PD-1 Therapy in Metastatic Melanoma. *Cell*. 2016;165(1):35-44.
10. Miao D, Margolis CA, Gao W, et al. Genomic correlates of response to immune checkpoint therapies in clear cell renal cell carcinoma. *Science*. 2018;359(6377):801-806.

Supplementary Figure 1. The comparison of survival probability between cancer patients with low (0-50%) and high (50%-100%) expression levels of *BLM*.

Supplementary Figure 2. The infiltration of multiple immune cells in cancer patients with low (0-50%) and high (50%-100%) expression levels of *BLM*.

Supplementary Figure 3. The composition of cancer types with or without *BLM* alteration in the cancer genome atlas database. ACC: adrenocortical carcinoma; AML: acute myeloid leukemia; BLCA: bladder urothelial carcinoma; BRCA: breast invasive carcinoma; BRCNOS: breast invasive carcinoma epithelial neoplasm; CCRCC: clear cell renal cell carcinoma; CEEN: cervical endometrioid carcinoma; CENU: mucinous carcinoma; CESC: cervical squamous cell carcinoma; CHOL: cholangiocarcinoma; CHRCC: chromophobe renal cell carcinoma; COAD: colon adenocarcinoma; DDLS: dedifferentiated liposarcoma; DES: desmoid/aggressive fibromatosis; DIFG: diffuse glioma; DLBCLNOS: diffuse large B-cell lymphoma, not otherwise specified; DSTAD: diffuse type stomach adenocarcinoma; ECAD: endocervical adenocarcinoma; EMBCA: embryonal carcinoma; ESCA: esophageal carcinoma; ESCC: esophageal squamous-cell carcinoma; FLC: fibrolamellar carcinoma; GBM: glioblastoma multiforme; HCC: hepatocellular carcinoma; HNSC: head and neck squamous cell carcinoma; IDC: invasive ductal carcinoma; IHCH: intrahepatic cholangiocarcinoma; ILC: invasive lobular carcinoma; IMMC: breast invasive mixed mucinous carcinoma; ISTAD: intestinal type stomach adenocarcinoma; LGGNOS: low-grade gliomas neoplasm; LMS: leiomyosarcoma; LUAD: lung adenocarcinoma; LUSC: lung squamous cell carcinoma; MACR: mucinous adenocarcinoma of the colon and rectum; MBC: metastatic breast cancer; MFH: malignant fibrous histiocytoma;

MFS: myxofibrosarcoma; MGCT: microinvasive germ cell tumor; MPNST: malignant peripheral nerve sheath tumor; MSTAD: mucinous stomach adenocarcinoma; OAST: oligoastrocytoma; ODG: oligodendroglioma; PAAD: pancreatic adenocarcinoma; PGNG: paraganglioma; PHC: pheochromocytoma; PHCH: perihilar cholangiocarcinoma; PLBMESO: pleural mesothelioma, biphasic type; PLEMESO: pleural mesothelioma, epithelioid type; PLSMESO: pleural mesothelioma; PRAD: prostate adenocarcinoma; PRCC: papillary renal cell carcinoma; PSTAD: papillary stomach adenocarcinoma; READ: rectum adenocarcinoma; SEM: seminoma; SKCM: skin cutaneous melanoma; SOC: serous ovarian cancer; SSRCC: signet ring cell carcinoma of the stomach; STAD: stomach adenocarcinoma; SYNS: synovial sarcoma; THPA: papillary thyroid cancer; THYM: thymoma; TSTAD: tubular stomach adenocarcinoma; TT: teratoma; TYST: yolk sac tumor; UCS: uterine carcinosarcoma; UEC: undifferentiated endometrial carcinoma; UM: uveal melanoma; UMEC: uterine mixed endometrial carcinoma; USC: uterine serous carcinoma

Supplementary Figure 4. The infiltration of multiple immune cells in cancer patients with different *BLM* copy numbers.

Supplementary Figure 5. The relationship between *BLM* copy number and the levels of tumor-infiltrating lymphocytes, immunomodulators (major histocompatibility complexes (MHCs), immunoinhibitors, immunostimulators), chemokines and chemokine receptors.

Supplementary Figure 6. Comparison of survival probability between immunotherapy-treated non-small cell lung cancer patients with low or high risk scores. The risk score was calculated

based on baseline age, Eastern Cooperative Oncology Group (ECOG) performance status, *EML4-ALK* rearrangement status, and *BLM* mutation. Patients were classified into two groups: low or high risk scores. The 'number at risk' table was shown below the Kaplan-Meier survival curve. HR: hazard ratio.

Supplementary Figure 1

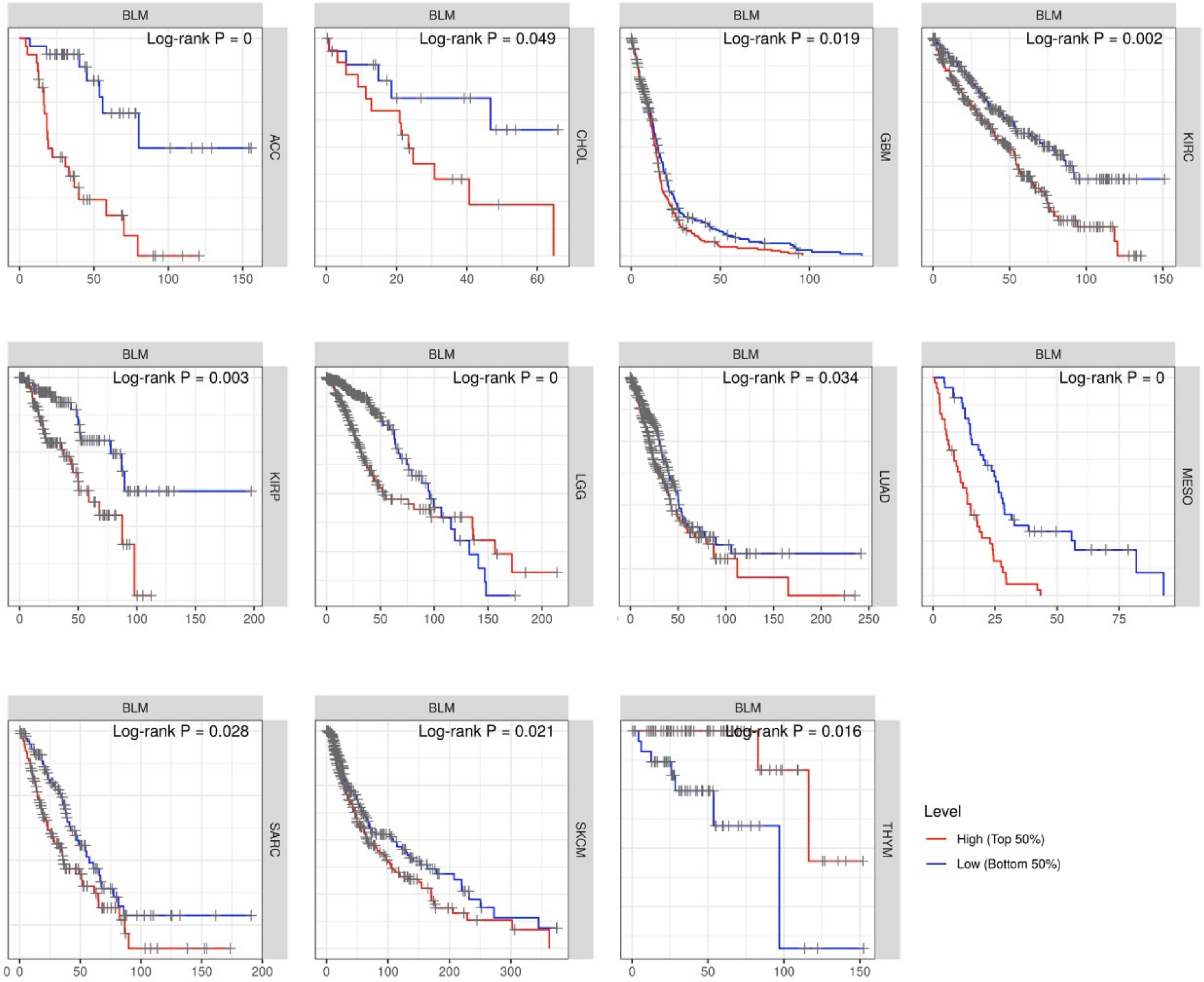

Supplementary Figure 2

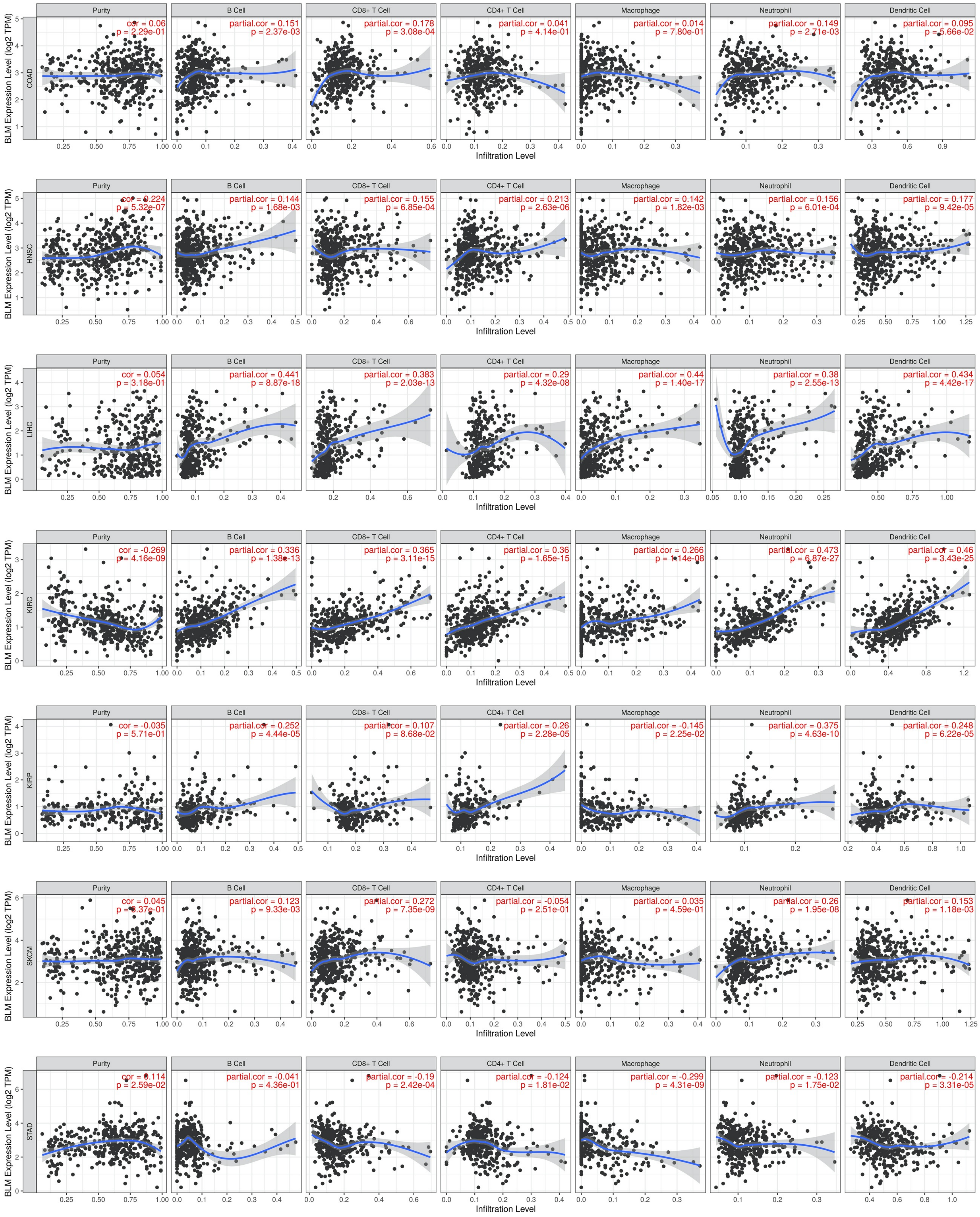

Supplementary Figure 3

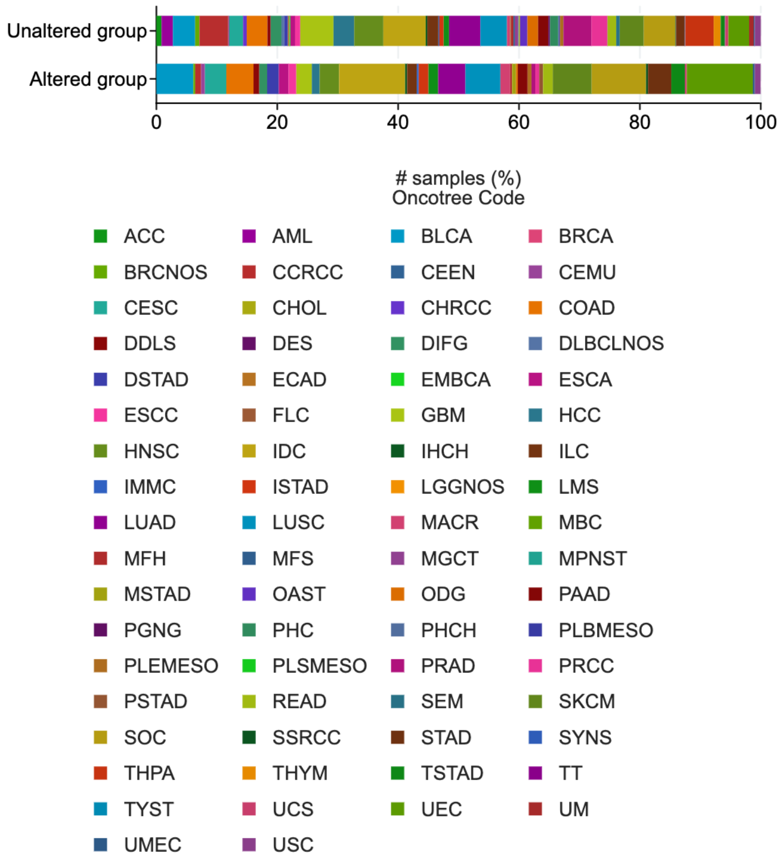

Supplementary Figure 4

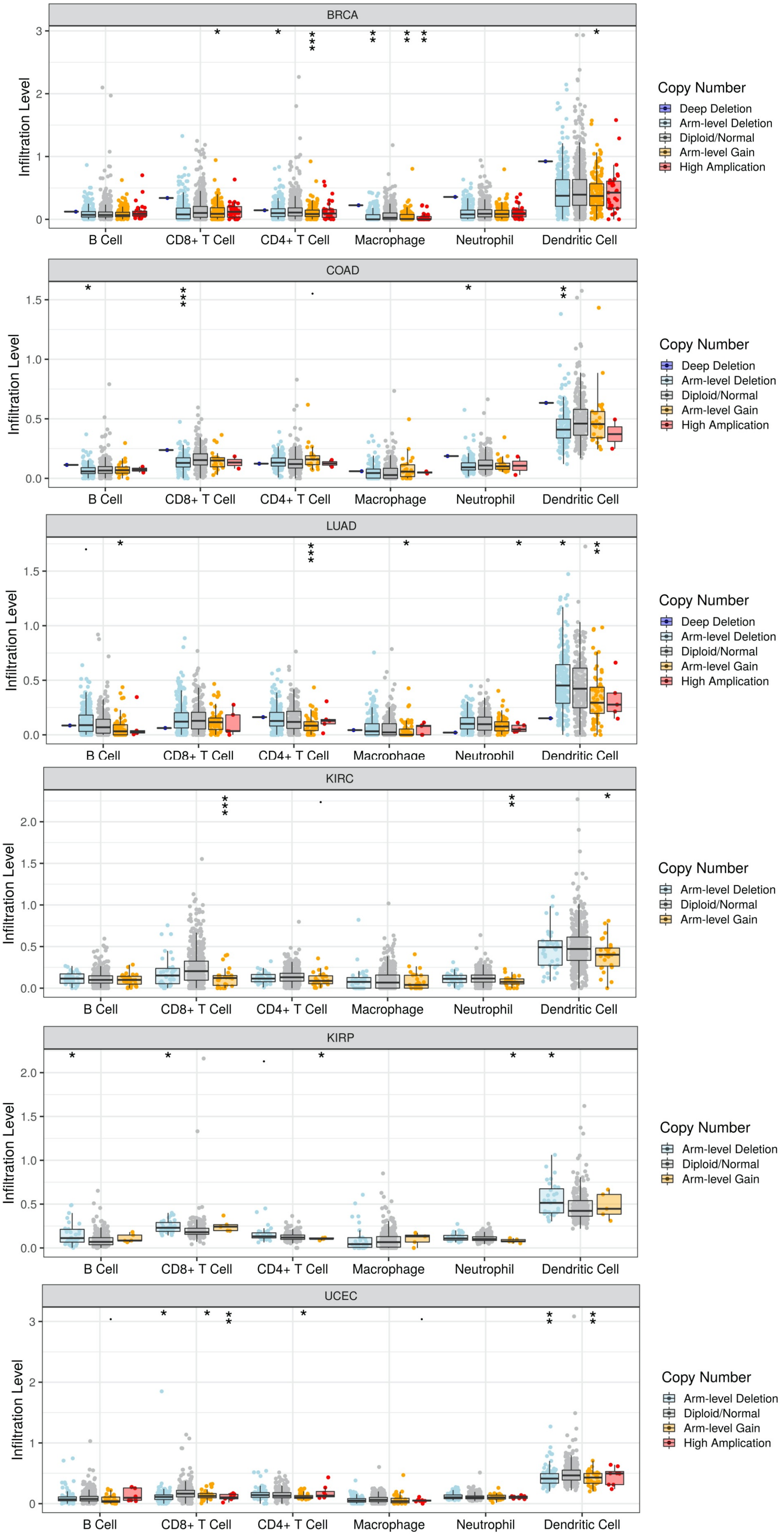

P-value Significant Codes: 0 ≤ *** < 0.001 ≤ ** < 0.01 ≤ * < 0.05 ≤ . < 0.1

Supplementary Figure 5

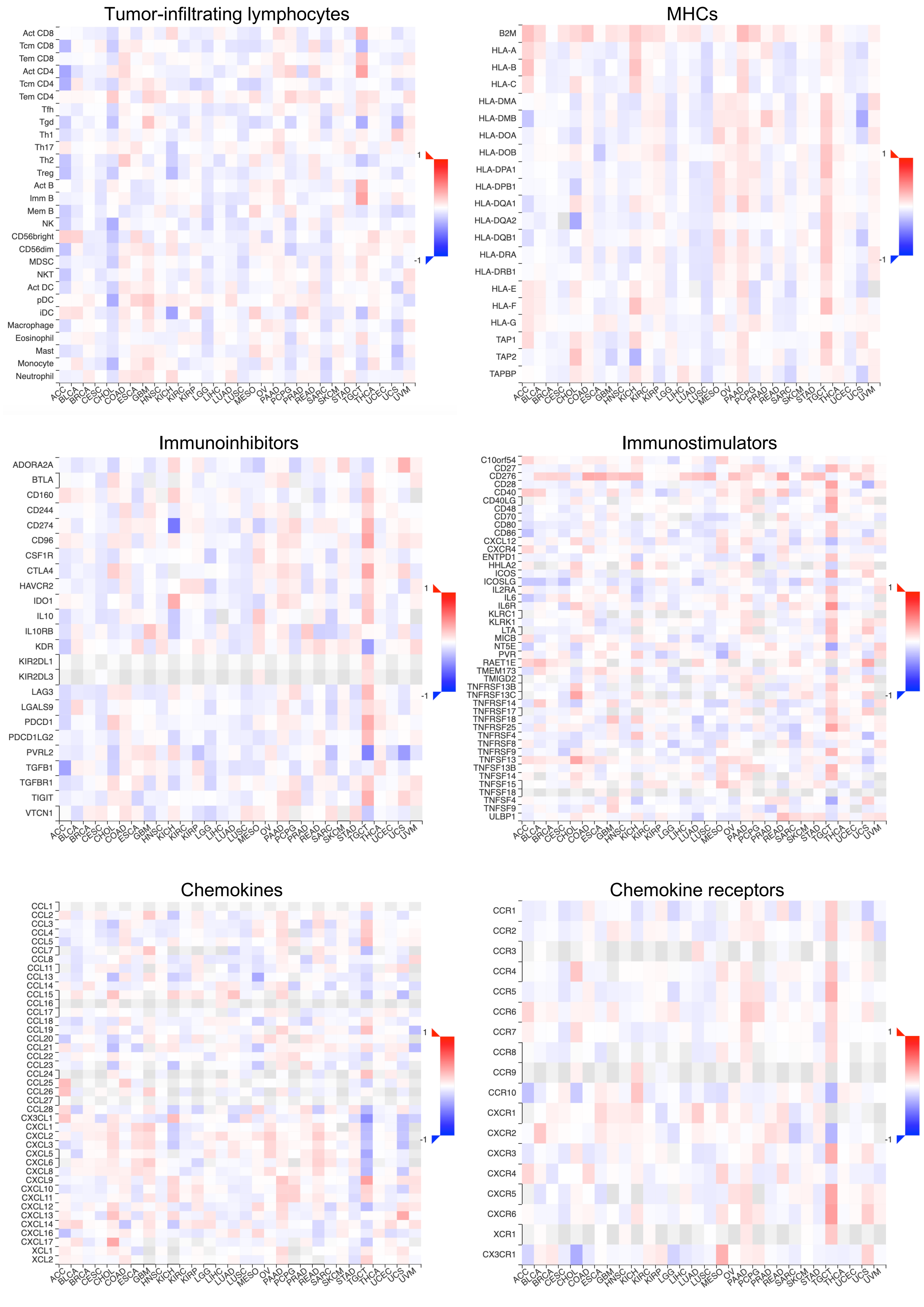

Supplementary Figure 6

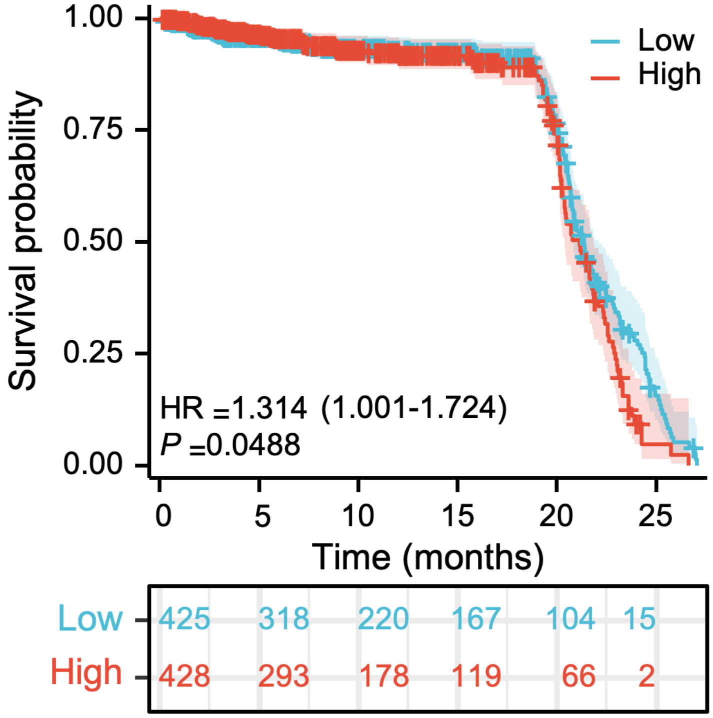

Supplement: Supplementary file 1 — Data S1. [file CAM4-13-e6716-s001.pdf]
